# Supplementary material for: Public awareness of seafood mislabeling
Source: PeerJ. 2022 Jun 28;10:e13486. doi: 10.7717/peerj.13486 (PMC9248779; doi:10.7717/peerj.13486)
Supplement: Supplemental Information 1 [file peerj-10-13486-s001.docx]

**Supplemental Material**

**Supplement 1.** Ten question survey given to respondents.

Hello! This is a fast and easy survey designed to assess awareness of seafood labeling practices and how important they are to seafood lovers.

This survey is conducted through the Department of Biology at the University of North Carolina at Chapel Hill. It should take about 5 minutes to complete. There are no correct or incorrect responses, and you may stop the survey at any time. You must be at least 18 years or older to participate. To protect your identity as a research subject, all responses to the survey will be anonymous. In any publication of this research, your name or other private information will not be used.

If you have any questions about this research, please contact John Bruno by calling 919-360-7651 or emailing jbruno@unc.edu. If you have questions or concerns about your rights as a research subject, you may contact the UNC Institutional Review Board at 919-966-3113 or by email to IRB_subjects@unc.edu.

Q1. How often do you consume seafood?

Very often (several times per week)

Often (once a week)

Sometimes (once or twice a month)

Seldom (once or twice a year)

Never

Q2. Where do you typically purchase your seafood? (check all that apply)

Seafood market

Grocery store

Restaurant

Other

I do not purchase seafood

Q3. How familiar are you with seafood mislabeling of seafood fraud?

Very familiar

Vaguely familiar

Never heard of it

Seafood mislabeling or fraud occurs when a seafood product is labeled under a different name than its actual species identity. For example, when farmed, imported shrimp is sold as “local, fresh” or when tilapia is sold as red snapper.

Q4. How concerned are you about seafood mislabeling?

Extremely concerned

Very concerned

Moderately concerned

Slightly concerned

Not at all concerned

Q5. Which issues related to mislabeling concern you? (check all that apply)

Health (allergies to certain types of seafood, mercury content, etc.)

Extremely concerned

Very concerned

Moderately concerned

Slightly concerned

Not at all concerned

Social justice issues (including seafood slavery)

Extremely concerned

Very concerned

Moderately concerned

Slightly concerned

Not at all concerned

Environmental concerns (such as endangered species being substituted for sustainably harvested species)

Extremely concerned

Very concerned

Moderately concerned

Slightly concerned

Not at all concerned

Economic (paying more for seafood than it’s actually worth)

Extremely concerned

Very concerned

Moderately concerned

Slightly concerned

Not at all concerned

Q6. About one third of all seafood is mislabeled. And for some species and vendor types, the percentage is much higher.

Knowing this, how likely would you be to preferentially purchase seafood from a restaurant or retailer where the labeling was independently verified?

Extremely likely

Somewhat likely

Neither likely nor unlikely

Somewhat unlikely

Extremely unlikely

Q7. Are you a student?

Yes

No

Q8. What is your year of birth?

Q9. In what country do you currently reside?

Q10. If you live in the US, in which state do you reside?

**
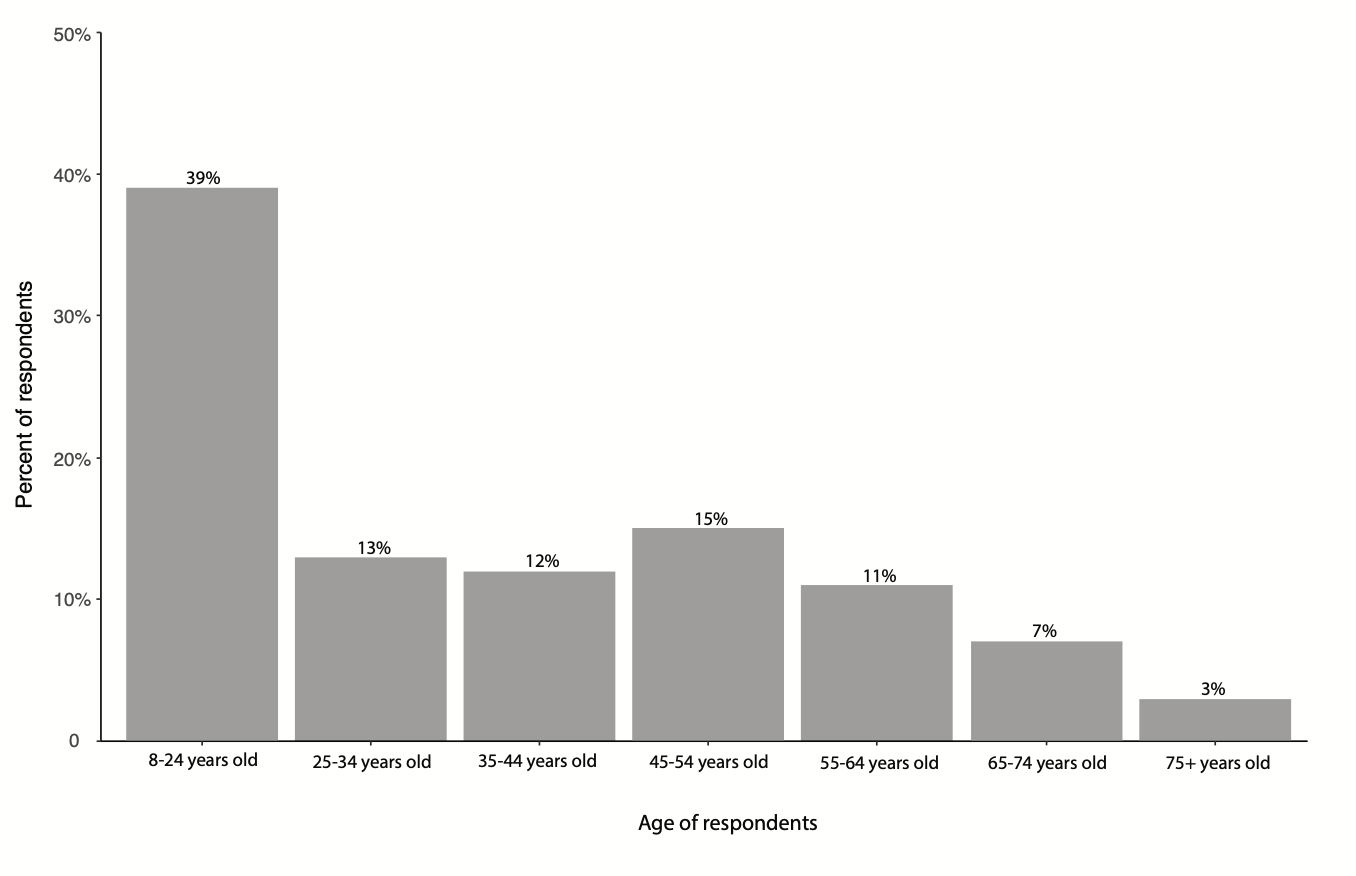
**

**Supplement 2.** Age distribution of survey respondents. Figure includes all responses (*n* = 1,216).

**Supplement 3.** Distribution of survey respondents based on the country in which they reside. Table includes all responses (*n* = 1,216).

| **Country of residence** | **Number of respondents** |
| --- | --- |
| Antigua and Barbuda | 1 |
| Argentina | 3 |
| Australia | 2 |
| Côte d'Ivoire | 3 |
| Canada | 3 |
| Cuba | 1 |
| Denmark | 1 |
| France | 1 |
| Germany | 1 |
| Italy | 1 |
| Mexico | 3 |
| Norway | 2 |
| Philippines | 1 |
| South Africa | 1 |
| Switzerland | 2 |
| United Arab Emirates | 1 |
| United Kingdom of Great Britain and Northern Ireland | 2 |
| United States of America | 1186 |
| Vietnam | 1 |

**Supplement 4.** Distribution of survey respondents based on the state in which they reside if they lived in the United States of America. Table includes respondents who answered that they lived in the USA; not all respondents who answered USA provided the state (*n* = 971).

| **State of residence** | **Number of respondents** |
| --- | --- |
| Alabama | 5 |
| Alaska | 2 |
| Arizona | 5 |
| California | 31 |
| Colorado | 9 |
| Connecticut | 9 |
| District of Columbia | 6 |
| Florida | 48 |
| Georgia | 27 |
| Hawaii | 3 |
| Idaho | 2 |
| Illinois | 8 |
| Indiana | 2 |
| Iowa | 2 |
| Kansas | 3 |
| Louisiana | 2 |
| Maine | 6 |
| Maryland | 12 |
| Massachusetts | 30 |
| Michigan | 5 |
| Minnesota | 41 |
| Missouri | 2 |
| Montana | 3 |
| Nevada | 1 |
| New Jersey | 13 |
| New Mexico | 1 |
| New York | 20 |
| North Carolina | 759 |
| North Dakota | 2 |
| Ohio | 8 |
| Oklahoma | 2 |
| Oregon | 1 |
| Pennsylvania | 25 |
| Rhode Island | 7 |
| South Carolina | 21 |
| Tennessee | 5 |
| Texas | 11 |
| Utah | 1 |
| Vermont | 2 |
| Virginia | 26 |
| Washington | 4 |
| West Virginia | 2 |
| Wisconsin | 8 |
| Wyoming | 1 |
